# Pneumothorax Following Acupuncture

**DOI:** 10.7759/cureus.14207

**Published:** 2021-03-31

**Authors:** Kirsten Weagle, Ryan J Henneberry, Paul Atkinson

**Affiliations:** 1 Emergency Medicine, Dalhousie University, Halifax, CAN; 2 Emergency Medicine, Saint John Regional Hospital, Saint John, CAN

**Keywords:** pneumothorax (ptx), point-of-care-ultrasound, ultrasound (u/s), thoracic imaging

## Abstract

Pneumothorax, the accumulation of air between the visceral and parietal pleurae, represents a potentially serious cause of chest pain in patients presenting to the emergency department. While there are known risk factors for spontaneous pneumothorax, in rare cases dry needling and acupuncture, forms of complementary and alternative medicine, have been known to result in pneumothorax.

We present a case of a 38-year-old healthy female who presented with acute onset of pleuritic chest pain to the Emergency Department. On further history, it was discovered that she had received dry needling acupuncture for unrelated back pain the day prior to presentation. She was initially investigated with an electrocardiogram (ECG), which was unremarkable, and point of care ultrasound (POCUS), which showed absent lung sliding and the presence of a lung point on the right side, indicative of pneumothorax. This case describes the key features of pneumothorax on POCUS and how POCUS can be a valuable tool in the diagnosis of lung pathologies and highlights the importance of considering the rare but potentially serious complications of complementary and alternative medicine practices which are typically regarded as safe.

## Introduction

In the emergency department (ED), chest pain is a common presenting complaint. The differential diagnosis of chest pain is broad and non-specific. Patients must be assessed for risk factors, signs, and symptoms that suggest or support a serious cause. Identifying the cause of chest pain is essential for timely and targeted interventions [1].

The use of point of care ultrasound (POCUS) by Canadian emergency physicians is well established [2]. POCUS can be used during the resuscitation of a critically ill patient, as an adjunct to history and physical examination in narrowing a differential diagnosis, and during procedures in the ED. Thoracic POCUS can be used in the identification of pneumothorax, hemothorax, pleural effusion, pulmonary edema, and interstitial lung syndrome [3]. Pneumothorax occurs when air accumulates between the parietal and visceral pleurae, resulting in a ventilation/perfusion mismatch [4, 5]. Risk factors for spontaneous pneumothorax include smoking, body habitus (tall, thin), underlying lung disease, including chronic obstructive pulmonary disease and infectious aetiologies such as P. Carinii pneumonia for secondary spontaneous pneumothorax, as well as thoracic trauma in iatrogenic and traumatic pneumothorax [6]. There are implications of untreated pneumothorax, which include hypoxia, tension pneumothorax, and cardiopulmonary failure [4].

Dry needling is a form of acupuncture, a commonly used complementary and alternative medical therapy. In addition to acupuncturists, it is also sometimes practiced by doctors, nurses, and physiotherapists with varying levels of training [5]. Though there is limited conclusive evidence for the practice, a review of Cochrane Collaboration database entries for acupuncture suggests that the therapy has a role in certain conditions, including in the treatment of musculoskeletal pain, headache, nausea, and vomiting [7]. Though not common, there are known serious adverse events related to acupuncture, including case reports of pneumothorax [8,9]. Adverse events have been found to be more likely with poor technique or in the absence of proper training [9].

In this case, we present a healthy young female who was diagnosed in the ED with a pneumothorax via POCUS following acupuncture treatment. This case illustrates an uncommon cause of pneumothorax and the utility of POCUS in making a timely diagnosis in an otherwise healthy population without risk factors.

## Case presentation

A 38-year-old female patient presented to the ED with a 12-hour history of sharp, pleuritic, right-sided chest pain. The pain was worse with movement and was not associated with any shortness of breath or respiratory symptoms. She was otherwise healthy and had no history of, or risk factors for coronary artery disease or venous thromboembolism. One day prior to her presentation, she had received dry-needling treatment through a physiotherapy clinic for an unrelated back pain.

**Figure 1:**
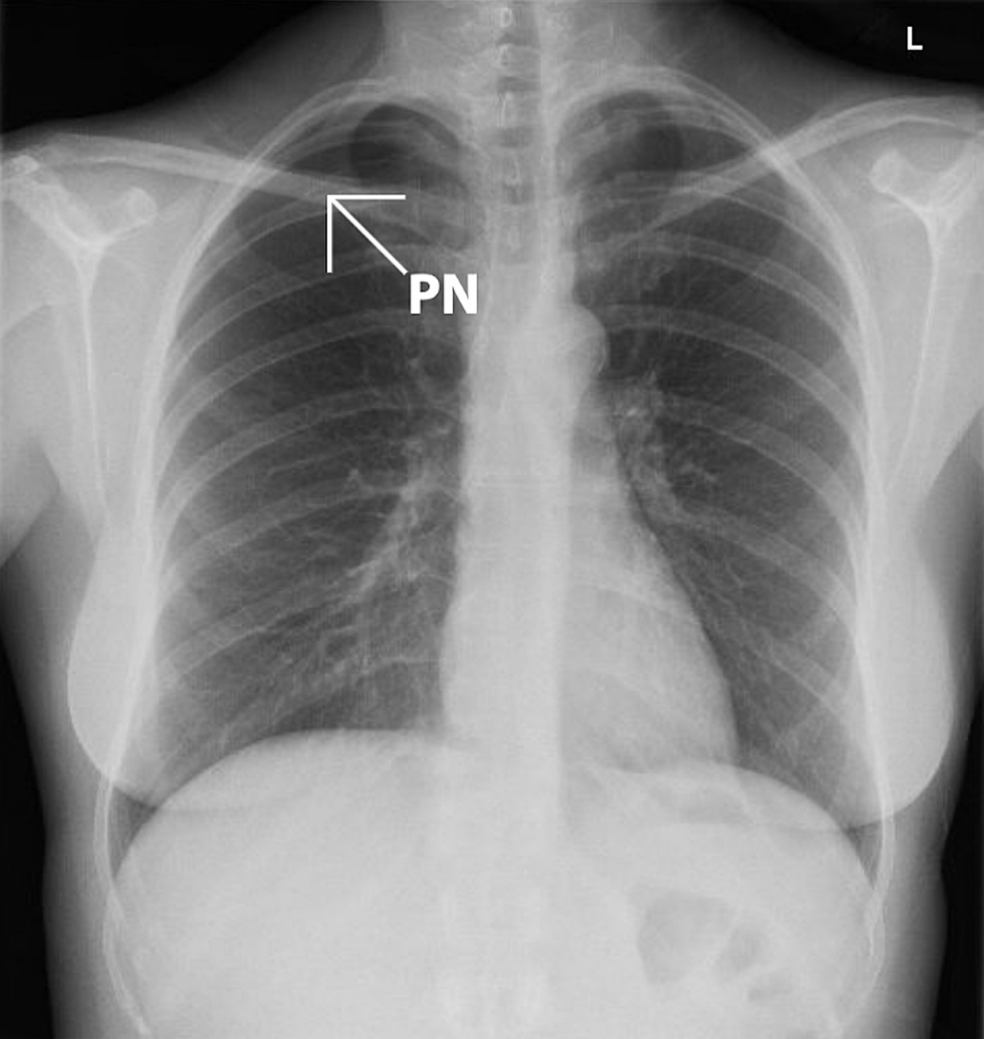
Posteroanterior (PA) chest X-ray demonstrating the presence of a right-sided pneumothorax (PN)

On presentation to the emergency department, she looked well. Her heart rate was 101, blood pressure was 105/69, respiratory rate was 18 and oxygen saturation was 98% on room air. Physical examination revealed slightly reduced air entry on the right with auscultation. Electrocardiogram (ECG) showed moderate voltage criteria for LVH and no ST changes. Cardiac enzymes were not measured. Point of care ultrasound (POCUS) was used to investigate a possible pneumothorax and revealed normal lung sliding on the left (Video 1), but absent lung sliding and the presence of a lung point on the right (Video 2). There was no evidence of other lung pathology or a pericardial effusion on ultrasound. A subsequent chest radiograph showed a small to moderate-sized right-sided pneumothorax with 2.2 cm of parietal pleural separation (Figure 1).

**Video 1:** POCUS in the longitudinal view of the left chest demonstrating normal lung sliding POCUS: Point of care ultrasound

**Video 2:** POCUS in the longitudinal view of the right chest demonstrating the lung point (arrow) indicating the presence of a pneumothorax POCUS: Point of care ultrasound

Using procedural sedation, a 9 French Cook catheter was placed in the right hemithorax. Placement was confirmed with a chest radiograph which also demonstrated re-expansion. The patient did have a transient episode of atrial flutter following chest tube placement. Her vital signs returned to normal and no treatment was required.

The patient returned to the ED two days after initial presentation for reassessment. Upon return, she was in no apparent respiratory distress. Her heart rate was 80, blood pressure 116/83, respiratory rate 20 and oxygen saturation 92% on room air. She had good air entry bilaterally and a repeat chest radiograph showed re-expansion with no evidence of pneumothorax. The chest tube was removed, and the site sutured with no further complications.

## Discussion

This case illustrates an occurrence where lung POCUS was used to expedite a diagnosis from a broad differential of life-threatening and common causes of chest pain. The patient had no underlying lung disease, nor any history of major thoracic trauma or other risk factors for a spontaneous pneumothorax (smoking or recreational drug use, family history, connective tissue disease, air travel, diving). Identification of pneumothorax with POCUS prompted confirmation with chest X-ray and further definitive management. While acupuncture is typically considered a safe procedure, adverse events can occur. An overview of systematic reviews of the safety of acupuncture found that pneumothorax was one of the most common types of organ or tissue injury resulting from acupuncture treatments [5].

Lung ultrasound (US) is a fast and easy way to potentially identify serious pathologies such as pneumothorax. Identifying the presence of lung sliding, a normal finding caused by the movement of the parietal and visceral pleura with inspiration, excludes pneumothorax [10]. In the presence of pneumothorax, air between the pleural layers prevents the visceral pleura from being visualized. At the border of the pneumothorax, where the pleural interface is intact, it is often possible to visualize a lung point which is definitive evidence of the presence of a pneumothorax [10].

While traditionally a radiologic diagnosis, there is data to show that ultrasound is both sensitive and specific in the diagnosis of a pneumothorax [11]. Identification of the lung point has been shown to be 100% specific. Studies have shown that diagnostic accuracy of ultrasound is in fact superior to chest radiograph and comparable to CT [11]. This suggests that the incorporation of POCUS in the evaluation of chest pain in the ED could reduce the need for chest radiographs [1]. POCUS allows the clinician to diagnose a pneumothorax with the additional advantages of avoiding radiation exposure, minimizing time to diagnosis, and avoiding transfer (for unstable patients) out of the ED.

## Conclusions

This case illustrates how POCUS can be a valuable tool in the diagnosis of potentially serious lung pathologies, emphasizing that emergency physicians should develop and maintain POCUS skills in this area. Emergency physicians should be aware of pneumothorax as a potential serious complication of complementary and alternative medical procedures such as dry needling and acupuncture, warranting consideration of this condition high in the differential diagnosis.
